# Concrete Incorporating a Spent CO_2_ Absorbent: Comprehensive Assessment of Microstructure, Strength, and Durability

**DOI:** 10.3390/ma19030577

**Published:** 2026-02-02

**Authors:** Sung-Lin Yang, Jong-Won Lee

**Affiliations:** Department of Highway & Transportation Research, Korea Institute of Civil Engineering and Building Technology, 283, Goyang-daero, Ilsanseo-gu, Goyang-si 10223, Republic of Korea

**Keywords:** CO_2_, DAC, CCUS, micro structure

## Abstract

Recycling spent CO_2_ absorbents generated from direct air capture (DAC) processes is important for improving the sustainability of carbon capture technologies. This study investigates the feasibility of using a spent alkaline CO_2_ absorbent as a partial replacement of mixing water in cementitious materials and evaluates its effects on microstructure, strength, and durability. Mortar and concrete mixtures were prepared with replacement ratios of 0–40%. Microstructural and phase evolution were analyzed using scanning electron microscopy, X-ray diffraction, and thermogravimetric analysis, while mechanical performance was assessed through compressive and flexural strength tests. Durability was evaluated by freezing–thawing resistance, chemical resistance in acidic environments, and accelerated carbonation tests. The results show that low replacement ratios (10–20%) improve early-age strength due to CaCO_3_-induced micro-filling and nucleation effects, while maintaining comparable long-term strength to the reference mixture. In contrast, higher replacement ratios (≥30%) cause excessive carbonation, C–S–H decalcification, increased micro-porosity, and strength reduction. Overall, spent CO_2_ absorbents can be effectively recycled in cementitious materials within a 10–20% replacement range.

## 1. Introduction

The global increase in carbon dioxide (CO_2_) emissions is accelerating climate change, and the resulting extreme weather events have emerged as a top priority for the international community. In response to these issues, the UN established a global cooperation framework by adopting the UN Framework Convention on Climate Change (UNFCCC) [1], and the 2015 Paris Agreement set a collective goal to limit the global average temperature increase to well below 2 °C above pre-industrial levels, and to pursue efforts to limit the increase to 1.5 °C [2]. Since then, each country has established reduction roadmaps and expanded CO_2_ emission reduction technologies in the energy and industrial sectors.

Recently, clean energy sources that minimize carbon emissions, such as solar, wind, and nuclear power, have rapidly expanded, and simultaneously, Carbon Capture, Utilization, and Storage (CCUS) technology has become a core strategy for mitigating greenhouse gas emissions from large-scale sources [3,4]. Conventional CCUS technology typically targets flue gases emitted from high CO_2_ concentration points, such as refinery processes or thermal power plants, and the captured CO_2_ is transported via pipeline or vehicle for utilization or permanent storage [5,6]. However, because these technologies are primarily applied to specific high-concentration emission sources, there is a limitation in fundamentally addressing the global CO_2_ accumulation problem [7].

To compensate for this limitation, Direct Air Capture (DAC) technology is emerging. DAC is a technology that directly separates low-concentration CO_2_ (approx. 0.04 vol% from the atmosphere without reliance on specific emission sources, offering fewer geographical constraints and being highly effective in removing residual CO_2_ that cannot be managed by conventional source-base CCUS technologies [8,9,10]. A DAC system consists of three stages: (1) contact between air and the sorbent, (2) CO_2_ capture through absorption or adsorption, and (3) sorbent regeneration and CO_2_ recovery. Since the partial pressure of CO_2_ in the atmosphere is only about 40 Pa [11], strong alkaline absorbents (such as Ca(OH)_2_, KOH, NaOH, and so forth) with high CO_2_ affinity are primarily used in the DAC process [11,12].

These strong alkaline absorbents have the advantage of being inexpensive and mass-producible, recovering CO_2_ in the form of carbonates (calcium carbonate, sodium carbonate, etc.) after reaction [7]. However, only approximately 20% of the sorbent used in the DAC system can be regenerated and reused, while the remaining 80% reacts with impurities during the CO_2_ capture process, leading to the problem of being classified as industrial waste [13,14]. Because the regeneration process also involves additional energy consumption and CO_2_ emissions [14], the importance of DAC sorbent recycling technology is gradually increasing. In this respect, the strategy of recycling spent sorbents into other industries becomes a critical solution that can enhance the circularity of DAC operation and offer an incidental carbon storage effect [15].

Specifically, the cement concrete sector is known to be a high-potential business area capable of accepting alkaline substances and carbonates. However, despite this characteristic, studies systematically analyzing the effects of directly applying spent DAC sorbents to cementitious materials on their strength, durability, and carbonation reaction are very limited.

Therefore, this study aims to evaluate the recyclability of the spent sorbent (recycled CO_2_ sorbent) generated from the DAC process by applying it as a replacement in cement mortar and concrete. By comprehensively reviewing the effects of the spent sorbent on the hydration reaction, carbonation process, microstructure, strength, and durability characteristics of cementitious materials, this research intends to provide fundamental data for resolving the issue of DAC sorbent disposal and for developing construction materials with CO_2_ storage capability.

## 2. Materials and Methods

### 2.1. Materials

#### 2.1.1. Binder

The binder used in this study consisted of ordinary Portland cement (OPC) complying with ASTM C150 [16] ground granulated blast-furnace slag (GGBS). The physical and chemical properties of the OPC and GGBS used in the experiment are summarized in Table 1 and Table 2.

#### 2.1.2. Spent CO_2_ Absorbent

The spent CO_2_ absorbent used in this study was an alkaline liquid absorbent collected from a DAC device after capturing approximately 99% of atmospheric CO_2_, at which point it could no longer be regenerated and was designated for disposal. The absorbent retained strong alkalinity after the capture cycle, exhibiting a measured pH of approximately 13.1. In this study, the spent CO_2_ absorbent was utilized in its original liquid form without any additional pretreatment or processing.

#### 2.1.3. ISO Graded Standard Sand

The sand consisted of rounded particles with a silicon dioxide content of 98% or more, and the particle size complied with the specification of KS L ISO 679 [18] Methods of testing cements—Determination of strength. The particle size distribution of ISO graded standard sand is shown in Table 3.

#### 2.1.4. Aggregate

In this study, fine aggregate with a particle size smaller than 5 mm was utilized, while coarse aggregate consisted of crushed granite with particle sizes ranging from 5 mm to 25 mm, sourced from Chungcheongnam-do Province in South Korea. The physical properties of the fine and coarse aggregates are presented in Table 4 and Table 5, respectively.

#### 2.1.5. Admixture

The high-range AE water-reducing agent of the poly carboxylic acid manufactured by “K” company in France was used as the admixture to improve the dispersion of cement and fine entraining of the air. Table 6 shows the physical properties of the admixture.

### 2.2. Mix Proportion and Preparation of Mortar Test Specimens

#### 2.2.1. Mix Proportion of Mortar

The mortar mixtures were designed in accordance with ISO 679 [19], using a binder-to-sand ratio of 1:3 and a water-to-binder ratio (W/B) of 50%. The spent CO_2_ absorbent was incorporated by replacing the mixing water at replacement ratios of 0%, 10%, 20%, 30%, and 40%. The detailed mix proportions are summarized in Table 7.

#### 2.2.2. Preparation of Mortar Specimens

To measure mortar compressive strength, test specimens of 40 mm × 40 mm × 160 mm were prepared according to ISO 679. After curing for 24 h in a constant temperature and moisture room, the mortar underwent removal of form followed by water curing at 20 °C. Compressive strength was measured for test specimens at varying ages.

#### 2.2.3. Microstructural and Chemical Characterization

The microstructural characteristics of the recycled CO_2_ sorbent–incorporated mortar were examined using scanning electron microscopy (SEM; secondary electron mode, 10–30 kV) to observe the morphology of hydration products and carbonation-induced microstructural changes. X-ray diffraction (XRD) analysis was conducted to identify crystalline phases such as portlandite, calcite, and aragonite and to evaluate phase evolution associated with CO_2_ replacement. Thermogravimetric analysis (TGA) was performed under a nitrogen atmosphere to identify characteristic mass-loss ranges associated with dehydration, portlandite decomposition, and CaCO_3_ decarbonation, allowing quantitative evaluation of the thermal stability and phase composition of the hydration and carbonation products.

#### 2.2.4. Mortar Test Method

The flow test was conducted after mixing of mortar in accordance with American Society for Testing and Materials (ASTM) Standards: C1437 Standard Test Method for Flow of Hydraulic Cement Mortar [20]. The flexural strengths and compressive strength tests were performed according to ISO 679, and measurements were taken at age 3, 7 and 28 days. A universal testing machine (UTM) of 100 tons was used to measure the compressive strength by age.

### 2.3. Mix Proportion and Preparation of Concrete Test Specimens

#### 2.3.1. Mix Proportion of Concrete

The concrete mixtures were designed with a target compressive strength of 30 MPa and a W/B of 48.6%. The binder consisted of OPC and GGBS, blended at a 50:50 ratio by mass. To evaluate the influence of the spent CO_2_ absorbent on concrete performance, the absorbent was incorporated as a partial replacement of mixing water at replacement ratios of 0% (Plain), 10%, and 20%. The detailed mix proportions are presented in Table 8. A polycarboxylic acid–based high-range water-reducing admixture was used to achieve a slump of 150 ± 25 mm and an air content of 4.5 ± 1.5%.

#### 2.3.2. Preparation of Concrete Specimens

Concrete specimens were prepared to evaluate the mechanical performance and durability of mixtures incorporating the Recycled CO_2_ Sorbent. Two specimen geometries were used depending on the required test: cylindrical specimens (100 mm in diameter and 200 mm in height) for compressive strength and chemical resistance evaluations, and beam specimens (100 × 100 × 400 mm) for flexural strength and accelerated carbonation tests. For each experimental program, approximately 20 specimens were fabricated to ensure statistical reliability and reproducibility of the results. After casting, all specimens were consolidated using an internal vibrator and stored for 24 h in a controlled-temperature chamber (20 ± 2 °C). Subsequently, the molds were removed, and the specimens were water-cured at 20 ± 2 °C until the designated test age.

#### 2.3.3. Compressive and Flexural Strength Test

The compressive strength was measured using a cylindrical specimen with dimensions of ϕ100 × 200 mm as per KS F 2405 “Test Method for Compressive Strength of Concrete” [21]. The specimen was tested using a 100-ton universal testing machine (UTM) after curing under water for 28 days at 20 ± 5 °C.

#### 2.3.4. Flexural Strength Test

The flexural Strength was measured using a rectangle specimen with dimensions of 100 × 100 × 400 mm as per KS F 2408 “Test Method for Flexural Strength for Concrete” [22]. The specimen was tested using a 100-ton universal testing machine (UTM) after curing under water for 28 days at 20 ± 5 °C.

#### 2.3.5. Freezing–Thawing Test

As for freezing–thawing tests, freezing–thawing was evaluated in accordance with method A (rapid underwater freezing–thawing tests) in KS F 2456 “Testing Method for Resistance of Concrete to Rapid Freezing–thawing” [23]. After curing in an underwater environment of 20 ± 2 °C up to the age of 14 days, the relative dynamic modulus of elasticity was measured every 30 cycles by the dynamic modulus of elasticity measurement device. was calculated by using the following Equation (1).

(1) $$p_{c}=\frac{{\left(n_{1}\right)}^{2}}{{\left(n\right)}^{2}}\times 100$$

where $P_{c}$: relative dynamic modulus of elasticity, after C cycles of freezing and thawing (%), n: fundamental transverse frequency at 0 cycles of freezing and thawing, and $n_{1}$: fundamental transverse frequency after C cycles of freezing and thawing.

#### 2.3.6. Chemical Resistance Test

Chemical resistance tests were performed in accordance with the test methods in ASTM C267-01 “Standard Test Method for Chemical Resistance of Mortars, Grouts, and Monolithic Surfacings and Polymer Concretes” [24]. After curing in a wet state at 20 ± 2 °C for 28 days, the specimens were immersed for 84 days in a 5% HCl solution and a 5% H_2_SO_4_, respectively. After 3, 7, 14, 28, and 56 days, the specimens were taken out, washed with tap water to remove the eroded and scaled parts, and dried in air to measure the mass change rate. was calculated using the following Equation (2).

(2) $$Mass\;loss\left(\%\right)=\frac{W-C}{C}\times 100$$

where C: conditioned weight of the specimen (g), W: weight of specimen after immersion (g).

#### 2.3.7. Accelerated Carbonation Test

The accelerated carbonation resistance tests were performed in accordance with KS F 2584, “Standard Test Method for Accelerated Carbonation of Concrete” [25]. The specimens were demolded after 24 h and cured in water for 4 weeks. After curing, they were stored for an additional 4 weeks in a constant temperature and humidity room with a relative humidity of 65 ± 5% and a temperature of 20 ± 2 °C. 

The specimens were then subjected to accelerated carbonation tests at a temperature of 20 °C, a relative humidity of 50%, and a CO_2_ concentration of 5%. At 1, 4, and 8 weeks, the specimens were split, and a 1% phenolphthalein solution was sprayed on the freshly fractured surface to measure the carbonation penetration depth.

## 3. Experiment Results and Analysis

### 3.1. SEM Result

**Figure 1:**
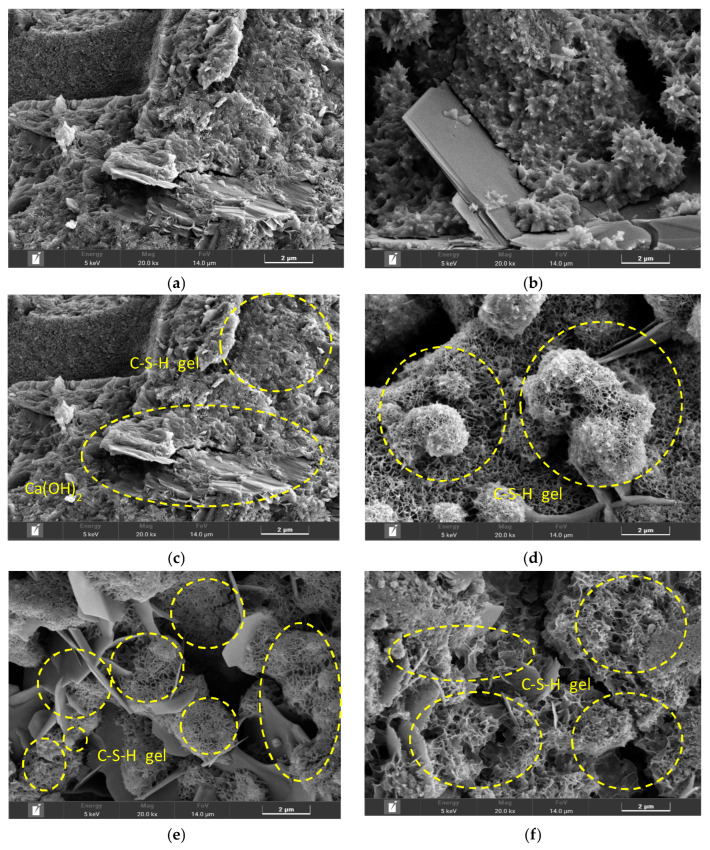
SEM images of mortar particles (**a**) Plain-1; (**b**) Plain-2; (**c**) CO_2_-10%; (**d**) CO_2_-20%; m (**e**) CO_2_-30%; (**f**) CO_2_-40%.

Figure 1 shows the microstructure characteristics of mortar with respect to the CO_2_ replacement ratio. The Plain specimen exhibited the typical morphology of mortar that underwent normal hydration, with residual ettringite structures (Figure 1a) and dense, amorphous C–S–H gel (Figure 1b) observed. Overall, the matrix maintained a dense and continuous form, suggesting a state with minimal external carbonation influence. In contrast, the CO_2_-replaced specimens (Figure 1c–f) showed distinct microstructural changes that may be associated with carbonation reactions.

As the CO_2_ content increased, CaCO_3_ precipitates were increasingly observed on the surface of the cement and slag hydration products, evolving from an initial fine flaky form to an increasingly agglomerated precipitated structure. While this CaCO_3_ formation appears to increase the apparent solid phase within the matrix, it should be noted that this observation is based on morphological features rather than direct compositional quantification. The CaCO_3_ formation is therefore interpreted as resulting not from new Ca ingress but from the conversion of existing Ca from C–S–H gel and Ca(OH)_2_ into carbonates via reaction with CO_2_ [26].

In cementitious hydrates, Ca(OH)_2_ directly reacts with CO_2_ to convert into CaCO_3_

Ca(OH)_2_ + CO_2_ → CaCO_3_

Furthermore, the degree of decalcification in slag-based C–S–H gel is expected to vary with CO_2_ concentration, with a larger fraction of Ca potentially converting to carbonate species as the CO_2_ content increases. This process can be conceptually described by the following reactions:

3CaO · 2SiO_2_ · 3H_2_O + CO_2_ → CaCO_3_ + 2CaO · 2SiO_2_ · 3H_2_O

3CaO · 2SiO_2_ · 3H_2_O + 2CO_2_ → 2CaCO_3_ + CaO · 2SiO_2_ · 3H_2_O

3CaO · 2SiO_2_ · 3H_2_O + 3CO_2_ → 3CaCO_3_ + 2SiO_2_ + 3H_2_O

As illustrated by these reaction schemes, progressive Ca depletion from C–S–H is theoretically expected with increasing CO_2_ exposure. Consequently, the formation of CaCO_3_ may be accompanied by the transformation of residual C–S–H into an SiO_2_-rich skeleton structure, which has been reported in previous studies to reduce gel stability [26,27]. Therefore, although an increased presence of CaCO_3_ is observed at higher CO_2_ replacement ratios, this phenomenon is discussed here as a plausible outcome of C–S–H decalcification, rather than a directly quantified change in Ca/Si ratio.

At CO_2_ replacement ratios of 30% or higher, SEM images show morphological features characterized by reduced apparent continuity of the C–S–H gel and the presence of localized porous regions. These features suggest a possible degradation of the original microstructural integrity, which may be associated with continuous Ca migration and consumption during the carbonation process [28].

### 3.2. XRD Result

**Figure 2:**
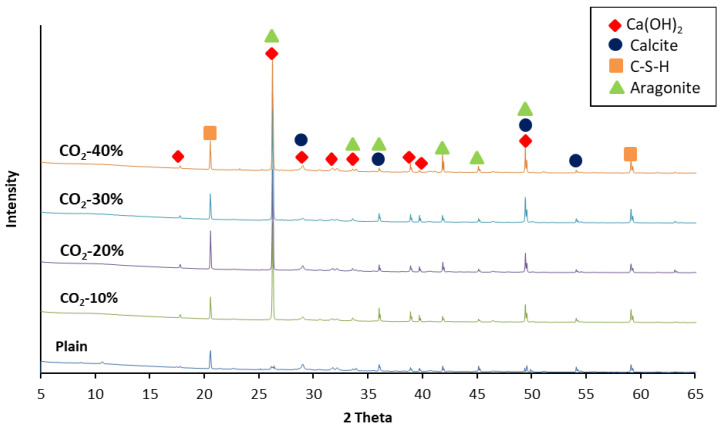
XRD spectra of mortar particles.

The XRD analysis results (Figure 2) clearly showed phase changes induced by carbonation reactions within the specimens as the CO_2_ incorporation ratio increased. In the Plain specimen, strong diffraction peaks corresponding to portlandite (Ca(OH)_2_, PDF No. 44-1481) were observed at 18°, 34°, 47°, and 50°, whereas the diffraction peaks associated with calcium carbonate phases, including calcite (CaCO_3_, PDF No. 05-0586) and aragonite (CaCO_3_, PDF No. 41-1475), were very minimal.

This limited presence of CaCO_3_ phases in the Plain specimen is attributed to slight natural carbonation during mixing, curing, and handling, the presence of carbonate impurities originating from ground granulated blast furnace slag (GGBS), and the partial decomposition of minor calcium-containing hydrates [29].

These results indicate that the hydration reaction proceeded normally in the Plain specimen, leading to well-developed portlandite and C–S–H gel as the primary binding phases.

In specimens with 10–20% CO_2_ incorporation, the intensity of the portlandite peaks (Ca(OH)_2_, PDF No. 44-1481) gradually decreased, while faint diffraction peaks corresponding to calcite (29.4°, 39.4°, and 47.5°; PDF No. 05-0586) and aragonite (26.2° and 27.2°; PDF No. 41-1475) began to appear. With 30% CO_2_ incorporation, these carbonate-related peaks became more distinct, suggesting that active carbonation of portlandite and partial carbonation of C–S–H occurred simultaneously [30]. This observation is consistent with previous studies reporting that accelerated carbonation environments favor the formation of aragonite.

For the specimen with 40% CO_2_ incorporation, the diffraction peaks of portlandite (PDF No. 44-1481) sharply diminished, while the peaks corresponding to aragonite (PDF No. 41-1475) and calcite (PDF No. 05-0586) distinctly increased and intensified, respectively. This behavior reflects the typical characteristics of high-concentration carbonation reactions, in which rapid carbonation promotes dominant aragonite precipitation, followed by partial transformation into the thermodynamically more stable calcite phase [31,32]. This interpretation is consistent with the TGA results, which showed increased mass loss in the 530–650 °C range associated with aragonite decomposition and a reduced proportion of mass loss above 650 °C corresponding to calcite decomposition.

### 3.3. TGA Result

**Figure 3:**
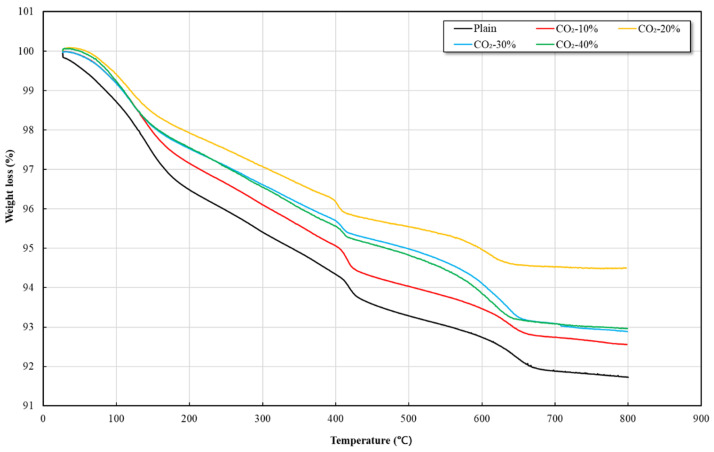
TGA Curves of mortar particles.

Figure 3 shows the thermogravimetric analysis (TGA) results for each mortar mix, confirming a distinct mass loss pattern separated into three temperature ranges across all specimens. The first range (approximately 100–150 °C) corresponds to the evaporation of free water and weakly bound adsorbed water, including the release of physically bound water from hydrates such as C–S–H gel and ettringite. The second range (approximately 400–430 °C) is mainly associated with the thermal decomposition of Ca(OH)_2_, and the mass loss in this range shows mix-specific variations depending on the degree of carbonation. The third range (approximately 500–670 °C) is generally associated with the decarbonation of carbonate-containing phases.

CaCO_3_ exhibits different thermal decomposition behaviors depending on its polymorphic form. Calcite, which is thermodynamically the most stable crystalline phase, typically decomposes at temperatures above approximately 650 °C, whereas aragonite decomposes over a relatively lower temperature range of approximately 530–650 °C. Vaterite, a metastable phase, is rarely observed under typical cementitious conditions due to its tendency to transform into aragonite or calcite at relatively low temperatures.

In the CO_2_-incorporated mortars investigated in this study, the mass loss observed in the 530–650 °C range was noticeably higher than that of the Plain specimen, while the relative contribution of mass loss at temperatures above 650 °C remained comparatively limited. This trend suggests that carbonate phases decomposing at lower temperatures were more pronounced in the CO_2_-incorporated specimens, although precise quantification of individual CaCO_3_ polymorphs cannot be achieved based on TGA data alone.

In the Plain specimen, a gradual mass loss was observed around approximately 600 °C. This behavior is attributed to the combined effects of multiple factors, including the decomposition of small amounts of CaCO_3_ formed through natural atmospheric carbonation, carbonate impurities originating from GGBS, and the high-temperature decomposition of certain hydration products. Na et al. [33] reported that hydrates such as C–S–H and C–(A)–S–H undergo dehydration and partial decomposition primarily above 600 °C, which is consistent with the gradual mass loss trend observed in the Plain specimen. Accordingly, the mass loss in this temperature range should be interpreted as a combined thermal response of impurity carbonates, amorphous calcium carbonate, and hydrate decomposition rather than as evidence of a dominant crystalline carbonate phase.

The CO_2_-incorporated specimens consistently exhibited increased mass loss in the 530–650 °C range, which is in agreement with previous studies reporting that aragonite-related carbonation products tend to decarbonate at relatively lower temperatures under accelerated carbonation conditions. Li et al. [34] and Yaseen et al. [35] reported that under high CO_2_ concentrations, partial carbonation of C–S–H occurs alongside Ca(OH)_2_ carbonation, often resulting in the initial formation of low-crystallinity aragonite, with subsequent transformation toward calcite over time. The mass loss in the 650–800 °C range, commonly associated with calcite decomposition, remained relatively small, indicating that high-temperature carbonate decomposition was less pronounced within the sensitivity limits of the present TGA, consistent with the findings of Madadi [36].

Overall, the TGA results indicate qualitative differences in the thermal decomposition behavior of carbonated phases between the Plain and CO_2_-incorporated specimens. These observations suggest that accelerated carbonation conditions favor the formation of carbonate phases that decompose at relatively lower temperatures; however, the relative proportions of individual CaCO_3_ polymorphs cannot be quantitatively resolved based on TGA data alone. The potential long-term transformation of these phases toward thermodynamically stable calcite remains consistent with existing literature [31,32].

### 3.4. Mortar Strength Test Result

The flowability of the fresh mortar was evaluated using the flow table test in accordance with ASTM C1437. The reference mixture (Plain) exhibited a flow diameter of 220 mm, indicating adequate workability. As the replacement ratio of the spent CO_2_ absorbent increased, the flow diameter gradually decreased to 200 mm and 190 mm for the CO_2_-10% and CO_2_-20% mixtures, respectively.

A more pronounced reduction in flowability was observed at higher replacement levels, with the flow diameter decreasing to 170 mm for CO_2_-30% and further to 160 mm for CO_2_-40%. This abrupt reduction beyond the 30% replacement level may be associated with the disruption of the glassy layer of GGBS, which can accelerate early-age hydration and heat evolution, thereby increasing the viscosity of the fresh mortar.

These results indicate that the spent CO_2_ absorbent significantly influences mortar workability and should be considered a key process parameter in mixture proportioning, particularly at higher replacement levels.

**Figure 4:**
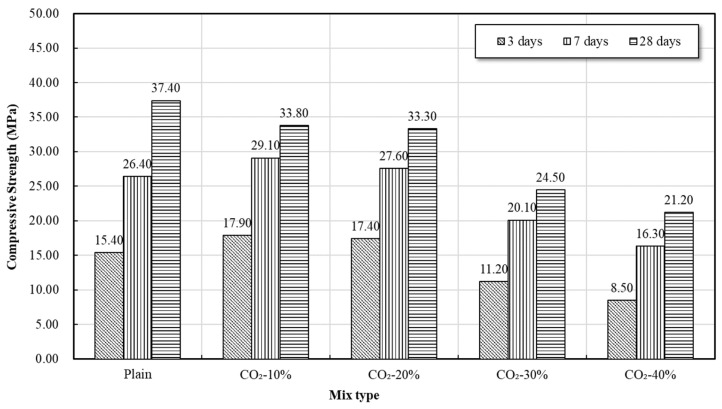
Compressive strength of mortar.

**Figure 5:**
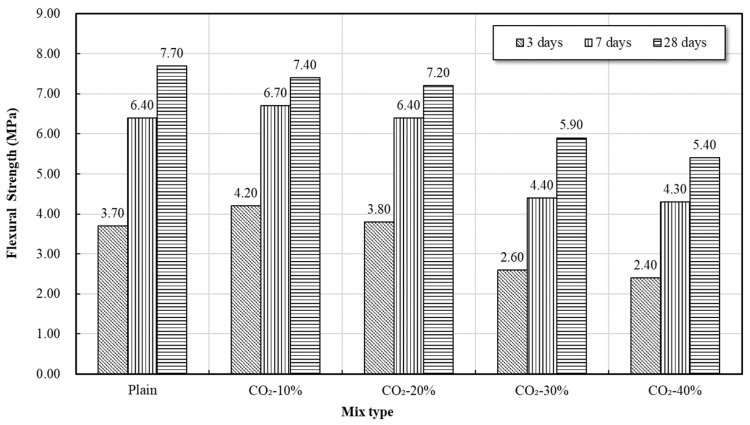
Flexural strength of mortar.

Figure 4 and Figure 5 show the change in compressive and flexural strength of mortar according to the CO_2_ replacement ratio. Overall, the strength tended to decrease as the CO_2_ replacement ratio increased, with a sharp drop in strength confirmed particularly at 30% or more CO_2_ replacement. Conversely, the 10% and 20% replacement mixes showed the characteristic of increased early-age strength (3-day and 7-day) compared to Plain.

The CaCO_3_ formed inside the mortar by CO_2_ injection simultaneously induces a carbonation reaction with the hydration reaction. Primarily, the two major hydrates, C–S–H (Calcium Silicate Hydrate) and Ca(OH)_2_ (Calcium Hydroxide), contribute to CaCO_3_ formation during the carbonation process. While some C_3_A (Calcium Aluminate) and C_4_AF (Calcium Aluminoferrite) can also react with CO_2_ to form CaCO_3_, their contribution to the total CaCO_3_ formation is known to be relatively small.

Specifically, the crystalline phase of CaCO_3_ varies depending on its origin: CaCO_3_ formed from Ca(OH)_2_ is generally calcite, while CaCO_3_ generated by the decalcification of C–S–H is typically formed as aragonite. Based on the XRD patterns and TGA thermal decomposition trends, the intensity of aragonite-related peaks and the mass loss in the 530–650 °C range increased with the CO_2_ replacement ratio, while the relative contribution of calcite-related decomposition at higher temperatures tended to decrease.

Aragonite tends to grow into needle-like crystals with higher aspect ratios than calcite, which has been reported to potentially disrupt matrix continuity and induce localized microstructural heterogeneity. This interpretation is consistent with the morphological features observed in the SEM images, such as reduced apparent continuity of the C–S–H gel and the presence of localized porous regions. Accordingly, when the CO_2_ replacement ratio exceeded 30%, the observed reduction in compressive and flexural strength may be associated with cumulative carbonation-related microstructural changes, including Ca(OH)_2_ consumption, partial decalcification of C–S–H, and the formation of carbonate phases decomposing at relatively lower temperatures.

On the other hand, a tendency of increased early-age strength compared to Plain was observed at CO_2_ replacement ratios of 10–20%, which is interpreted as a result of the combined physical and chemical effects of carbonation byproducts in a low-concentration CO_2_ environment.

First, the fine CaCO_3_ crystals formed through the initial carbonation reaction exert a micro-filling effect by partially filling the pores of the mortar, thereby inducing densification of the matrix. This micro-filling action not only reduces porosity but also contributes to stabilizing the initial load transfer path by increasing the interfacial contact between the C–S–H gel and CaCO_3_ particles.

Second, in the early stages of CO_2_ introduction, the temporary rise in pH, accompanied by the formation of carbonate species (HCO_3_^−^, CO_3_^2−^) in the solution, acts as a factor that increases the alkali activation of GGBS. Activated slag forms hydrates such as C–A–S–H more rapidly than conventional OPC-based hydration, accelerating the initial hydration reaction, which consequently improves the early-age strength development.

Third, the fine CaCO_3_ particles formed during carbonation function as nucleation sites for the formation of hydration products. The CaCO_3_ surface provides a foundation for the early growth of C–S–H, simultaneously increasing the rate and amount of C–S–H formation during the initial hydration stage, which plays a crucial role in enhancing early-age strength. This nucleation effect was particularly noticeable as a significant increase in 3-day and 7-day strength compared to Plain, demonstrating that CaCO_3_ acts not merely as a carbonation byproduct but also as an accelerator for the initial hydration reaction.

While these effects led to higher early-age strength in the 10% and 20% CO_2_-incorporated mortars compared to Plain, at 30% or more, where carbonation progressed excessively, the combined influence of these factors appears to contribute to strength reduction.

In summary, the mortar test results indicate that 10–20% CO_2_-incorporation is favorable for enhancing early-age strength, whereas CO_2_ replacement levels of 30% or higher are associated with pronounced strength reduction, likely due to carbonation-induced microstructural alterations. Accordingly, in the concrete production stage of this study, 10% and 20% CO_2_ replacement mixtures were selected for subsequent strength and durability tests.

### 3.5. Concrete Strength Test Result

**Figure 6:**
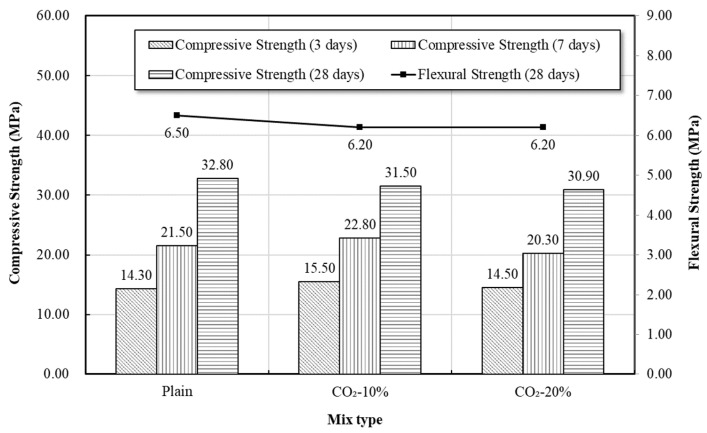
Compressive and Flexural strength of concrete.

Figure 6 shows the age-dependent compressive strength and 28-day flexural strength of concrete according to the CO_2_ replacement ratio (0%, 10%, 20%). Overall, the CO_2_-replaced concrete showed superior performance in early-age strength (3-day and 7-day) compared to Plain, similar to the trend observed in the mortar.

The CO_2_-10% and CO_2_-20% mixtures showed higher values of 15.5 MPa and 14.5 MPa in 3-day strength, respectively, compared to Plain (14.3 MPa), and the same trend was maintained in 7-day strength. This early-age strength enhancement may be associated with mechanisms similar to those observed in mortar, including early densification due to fine CaCO_3_ formation in a low-concentration CO_2_ environment, enhanced slag reactivity, and the CaCO_3_-based hydrate nucleation effect. However, due to the aggregate–mortar composite structure of concrete, it does not respond as sensitively as mortar to strength development, and thus the increase in early-age strength was relatively gradual.

In 28-day compressive strength, all three mixes showed similar levels of approximately 30 MPa (Plain: 32.8 MPa, CO_2_-10%: 31.5 MPa, CO_2_-20%: 30.9 MPa), and the reduction in long-term strength due to CO_2_ replacement was not significant. This is likely due to the structural complementary effect between the aggregate skeleton and the matrix in concrete, which mitigates strength reduction even when Ca(OH)_2_ is partially consumed and C–S–H is partially decalcified by CO_2_ replacement.

Meanwhile, the 28-day flexural strength did not show a large difference across all mixes, but the CO_2_-replaced mixes exhibited slightly lower values compared to Plain. This observation may be related to the fact that tensile crack resistance under flexural loading is more sensitive to local matrix continuity and microstructural homogeneity, and that the CaCO_3_ formed during the initial carbonation process, while contributing to matrix densification under compression, could locally influence crack propagation behavior.

In summary, concrete incorporated with CO_2_ within the 10–20% range showed superior performance in early-age strength compared to Plain, and maintained a similar level of long-term strength. This implies that low-concentration CO_2_ incorporation is positive for early strength development in concrete, and the tendency of structural damage observed in high-concentration replacement (based on mortar) is relatively mitigated in concrete.

### 3.6. Freezing–Thawing Test Result

**Figure 7:**
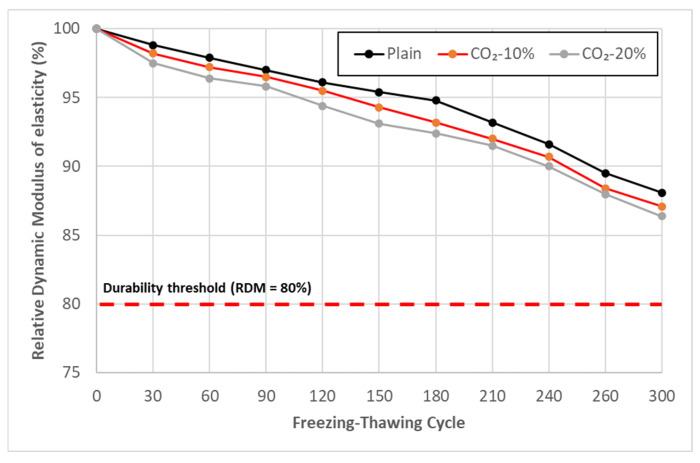
Relative dynamic modulus of elasticity versus number of freezing–thawing cycle.

Figure 7 shows the results of the freezing–thawing test performed for 300 cycles according to ASTM C666 Procedure A. The relative dynamic modulus of elasticity (RDM) criterion of 80% or more was stably satisfied for all mixes, and the CO_2_-10% and CO_2_-20% mixes showed no significant performance degradation in terms of freezing–thawing resistance compared to Plain.

The comparable freezing–thawing performance of the CO_2_-replaced mixes to Plain may be related to factors associated with the pore structure and saturation level of the concrete.

First, the fine CaCO_3_ particles generated through the initial carbonation reaction during low-concentration CO_2_ replacement are likely to contribute to subtle pore refinement by partially filling capillary pores within the concrete. Such pore refinement may help mitigate internal stress development during freezing by limiting water mobility, thereby reducing damage accumulation during freezing–thawing cycles. Furthermore, the CaCO_3_ crystals formed by CO_2_ replacement, which exhibit relatively high density and stability, could influence the distribution and mobility of free water within the concrete, potentially mitigating repetitive volumetric expansion during freezing and thawing. Frost resistance is largely governed by factors such as saturation degree, air content, and pore size distribution. The CO_2_ replacement ratio (10–20%) applied in this study did not appear to induce critical changes in these governing parameters, resulting in freezing–thawing resistance comparable to that of Plain.

Consequently, the RDM reduction trends of the CO_2_-replaced mixes were similar to those of Plain, suggesting that CO_2_ incorporation at the investigated replacement levels does not adversely affect freezing–thawing resistance.

### 3.7. Chemical Resistance Test Result

**Figure 8:**
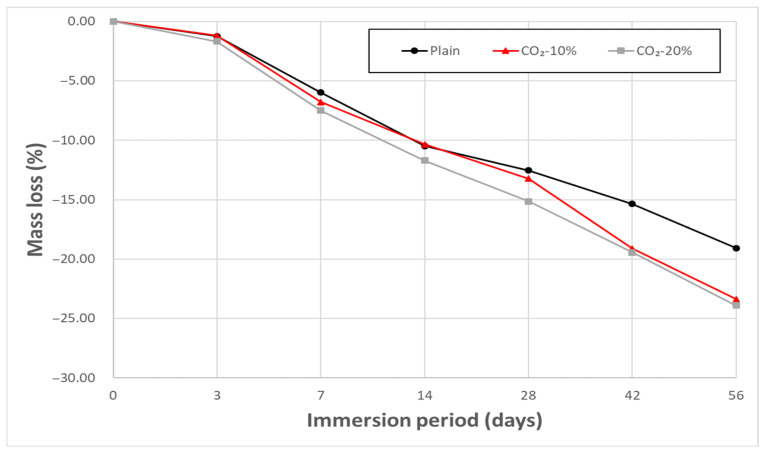
Mass loss of porous concrete after immersion in 5% HCl.

**Figure 9:**
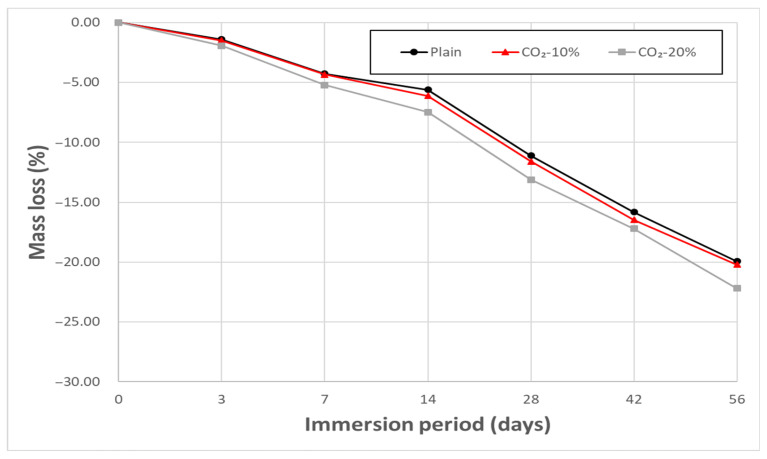
Mass loss of porous concrete after immersion in 5% H_2_SO_4_.

Figure 8 and Figure 9 show the mass loss trend of concrete immersed in 5% HCl and 5% H_2_SO_4_ solutions, respectively. In both tests, mass loss progressively increased across all mixes with increasing immersion time, consistent with the typical acid attack mechanism where major alkaline hydration products like Ca(OH)_2_ and C–S–H dissolve and surface spalling occurs in strong acid environments [37,38].

Notably, CO_2_-replaced concrete has an increased CaCO_3_ content due to prior carbonation, and the CaCO_3_ itself can react directly with the acid, further accelerating damage. In the HCl environment, CaCO_3_ dissolves rapidly, producing CaCl_2_ and CO_2_ through the following reaction:

CaCO_3_ + 2HCl → CaCl_2_ + H_2_O + CO_2_↑

The pre-carbonated CO_2_-replaced mixes may experience enhanced acid dissolution reactions compared to Plain, as CaCO_3_ also reacts with HCl and H_2_SO_4_ to form CaCl_2_ or CaSO_4_. This reaction is known to proceed rapidly, which may contribute to the relatively higher mass loss observed in CO_2_-replaced mixes in the HCl environment. Indeed, the Plain mix showed a 19.6% mass loss after 56 days of immersion, while the CO_2_-replaced mixes showed a loss of approximately 23.5%, suggesting a higher susceptibility of CO_2_-replaced concrete to HCl attack under the tested conditions.

The CO_2_ sorbent used in this study is primarily composed of a Na-based alkaline solution, making it likely that CO_2_-replaced concrete contains a higher Na^+^ content compared to Plain [39,40]. These Na^+^ ions do not significantly affect structural performance under normal conditions, but may readily dissolve under acidic environments, forming NaCl or Na_2_SO_4_ [41,42]. These highly soluble salts could act as secondary contributors to degradation by increasing ionic concentration within the pore solution, thereby facilitating leaching of hydration products and surface scaling [43].

In the 5% H_2_SO_4_ immersion test of Figure 9, continuous mass loss was also observed in all mixes, which is governed by the characteristic sulfate conversion reaction seen in sulfuric acid attack. H_2_SO_4_ first reacts with Ca(OH)_2_ to produce gypsum (CaSO_4_·2H_2_O), and subsequently reacts with C3A to form expansive ettringite (C3A·3CaSO_4_·32H_2_O):

Ca(OH)_2_ + H_2_SO_4_ → CaSO_4_·2H_2_O

C_3_A + 3CaSO_4_ + 32H_2_O → C_3_A·3CaSO_4_·32H_2_O

Moreover, CaCO_3_ also reacts with sulfuric acid to convert to CaSO_4_ and release CO_2_:

CaCO_3_ + H_2_SO_4_ → CaSO_4_ + CO_2_↑ + H_2_O

Because CO_2_-replaced concrete contains a higher CaCO_3_ content, additional CaSO_4_ can be formed through the above reaction, which may contribute to faster sulfate accumulation and associated expansive damage compared to Plain. Additionally, residual Na^+^ may react with sulfuric acid to form Na_2_SO_4_, potentially increasing sulfate concentration within the pore system and intensifying sulfuric acid attack. In practice, the CO_2_–20% mix showed a mass loss of 22.2% after 56 days of immersion, indicating higher degradation susceptibility than Plain (19.9%), while the CO_2_–10% mix maintained a similar level (20.2%) to Plain.

In summary, the chemical resistance of CO_2_-replaced concrete is governed by the combined effects of acid dissolution reactions and salt formation. Within the 10–20% CO_2_ replacement range, the overall degradation level was similar to Plain, indicating no pronounced adverse effect on structural stability under the conditions investigated.

### 3.8. Accelerated Carbonation Test Result

**Figure 10:**
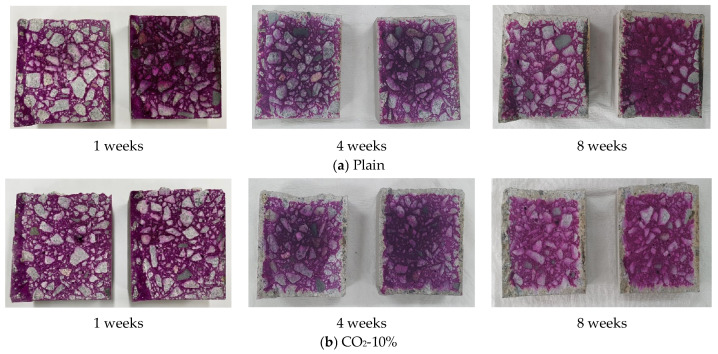

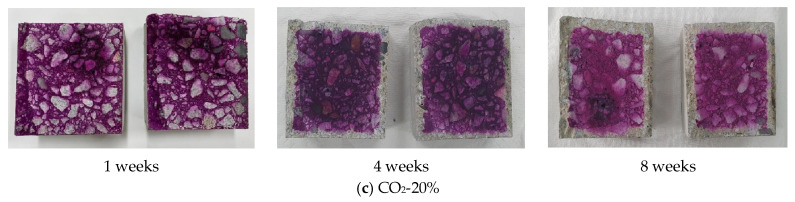
Result of penetration depth measurement (**a**) Plain; (**b**) CO_2_-10%; (**c**) CO_2_-20%.

Figure 10 shows the results of the accelerated carbonation test for each mix, where the carbonation penetration depth progressively increased in all specimens with increasing age. At 1 week, the entire surface of all mixes remained red after the application of the phenolphthalein indicator, indicating that sufficient Ca(OH)_2_ was present to maintain a high-pH environment during the early hydration stage [44].

Carbonation became apparent in all mixes after 4 weeks, and the average carbonation depth increased in the order of CO_2_–20% > CO_2_–10% > Plain. At 8 weeks, the measured carbonation depths were 7.27 mm for the CO_2_–20% mix, 5.80 mm for the CO_2_–10% mix, and 4.90 mm for Plain, showing a tendency for carbonation resistance to decrease with increasing CO_2_ replacement ratio. This tendency is qualitatively consistent with the microstructural observations obtained from SEM, XRD, and TGA analyses in this study.

In the CO_2_-replaced mixes, the microstructure may be more susceptible to carbonation due to pre-existing carbonation, which reduces the residual Ca(OH)_2_ content and is accompanied by partial decalcification of the C–S–H gel. Such microstructural changes have been reported to influence the diffusion and penetration behavior of the carbonation front [34,45]. Thiéry et al. [46] also reported that carbonation reaction rates increase under higher CO_2_ concentrations, which is consistent with the relatively faster carbonation progress observed in the CO_2_–20% mix in this study.

Previous studies have further indicated that while initial CaCO_3_ formation at low CO_2_ replacement levels may provide a micro-filling effect, excessive replacement can potentially disturb the C–S–H gel structure and reduce long-term chemical stability [47]. The characteristics of GGBS-containing systems are also relevant to carbonation susceptibility. According to Lothenbach et al. [48], GGBS reduces Ca(OH)_2_ formation and produces C–S–H with a lower Ca/Si ratio, which may increase vulnerability to carbonation. In this study, the CO_2_-replaced mixes exhibited slightly lower strength than Plain, particularly at the 20% replacement level, which could be associated with increased pore connectivity and permeability, thereby facilitating CO_2_ diffusion.

Taken together, the observed increase in carbonation depth in CO_2_-replaced concrete may be associated with a combination of reduced Ca(OH)_2_ availability, partial decalcification of C–S–H, and pore structure characteristics, especially in GGBS-containing mixes. These factors may contribute to the accelerated advancement of the carbonation front, resulting in a slight reduction in carbonation resistance compared to Plain.

Nevertheless, when the CO_2_ replacement ratio was limited to 10%, the carbonation depth remained comparable to that of Plain, suggesting that durability performance was not critically compromised under the conditions investigated. In contrast, at a 20% replacement level, the carbonation rate increased more noticeably, indicating that careful consideration is required for long-term durability when higher CO_2_ replacement ratios are applied.

## 4. Conclusions

This study comprehensively analyzed the microstructure, strength, and durability characteristics of mortar and concrete specimens partially incorporating spent CO_2_ absorbent, which is generated during the DAC process, as a preliminary investigation for recycling the waste absorbent into cementitious materials.

Key findings are as follows:

(1) CO_2_ absorbent replacement was observed to be associated with changes in the microstructure, including increased CaCO_3_ formation due to carbonation and partial decalcification of the C–S–H gel. At replacement ratios of 10–20%, CaCO_3_-related micro-filling and nucleation effects were found to coincide with a relatively dense pore structure. In contrast, at replacement levels of 30% or higher, SEM observations indicated a higher presence of localized micro-pores and reduced apparent continuity of the gel structure.
(2) Strength evaluation showed that CO_2_ absorbent replacement at 10–20% was associated with enhanced early-age strength compared to the Plain mixture. This behavior may be related to carbonate-based nucleation effects and the activation of GGBS in the presence of residual alkalinity. Conversely, replacement levels of 30% or higher were accompanied by reductions in compressive and flexural strength, which may be associated with carbonation-related microstructural changes, including partial C–S–H decalcification.
(3) In the durability assessment, CO_2_-replaced concrete exhibited freezing–thawing resistance comparable to that of the Plain mixture. Accelerated carbonation tests showed a tendency for carbonation resistance to decrease with increasing CO_2_ replacement ratio; however, at replacement levels of 10–20%, the carbonation depth remained within a range similar to that of Plain. These results suggest that, within this replacement range, durability performance was not critically compromised under the conditions investigated.
(4) Chemical resistance to strong acid solutions (5% HCl and 5% H_2_SO_4_) was slightly lower for CO_2_-replaced concrete compared to Plain. This tendency may be associated with acid dissolution reactions involving CaCO_3_, as well as the formation of soluble salts such as CaCl_2_, CaSO_4_, NaCl, and Na_2_SO_4_ originating from carbonation products and Na^+^ ions in the spent absorbent. Nevertheless, the increase in mass loss remained within a limited range, and mixtures with 10–20% replacement maintained a practically acceptable level of chemical resistance.
(5) Overall, the experimental results indicate that the spent CO_2_ absorbent can be applied to cementitious materials within a 10–20% replacement range without significant deterioration in mechanical or durability performance. This replacement range may offer potential benefits, including improved early-age strength, pore structure refinement, and the incorporation of carbonated products. While the present findings demonstrate the feasibility of recycling spent DAC absorbents into cementitious materials, further studies are required to quantitatively verify CO_2_ storage capacity and to evaluate long-term durability under field-relevant exposure conditions.

## Figures and Tables

**Table 1:** Physical and chemical properties of OPC [17].

Density (g/cm^3^)	Fineness (cm^2^/g)	Chemical Properties (%)						
		SiO_2_	Al_2_O_3_	Fe_2_O_3_	CaO	MgO	SO_3_	Ig.loss
3.14	3492	21.1	4.65	3.14	62.8	2.81	2.1	2.18

**Table 2:** Physical and Chemical properties of GGBS.

Density (g/cm^3^)	Blaine Fineness (cm^2^/g)	Chemical Properties (%)						
		SiO_2_	Al_2_O_3_	Fe_2_O_3_	CaO	MgO	SO_3_	Ig.loss
2.90	4570	24.7	16.4	0.18	49.1	2.73	1.52	0.68

**Table 3:** Particle size distribution of ISO graded standard sand [18].

Sieve size (mm)	2.0	1.6	1.0	0.5	0.16	0.08
Cumulative passing (%)	0	7 ± 5	33 ± 5	67 ± 5	87 ± 5	99 ± 5

**Table 4:** Physical properties of fine aggregate [17].

Density (g/cm^3^)	Absorption (%)	Unit Weight (kg/m^3^)	Fineness Modulus	Sound (%)
2.59	1.08	1598	2.75	2.7

**Table 5:** Physical properties of coarse aggregate [17].

Density (g/cm^3^)	Absorption (%)	Unit Weight (kg/m^3^)	Fineness Modulus	Sound (%)
2.70	1.82	1566	7.05	3.1

**Table 6:** Physical properties of admixtures.

Appearance	Density (g/cm^3^)	pH	Active Matter (%)
Brownish powder	0.37	6.0 ± 1	98 ± 2

**Table 7:** Mix proportion of Mortar.

Test ID		W/B (%)	Mix Composition (g)				
			Sand	OPC	GGBS	Water	Spent CO_2_ Absorbent (aq.)
Plain		50	1350	225	225	225	-
CO_2_	10%					202.5	22.5
	20%					180.0	45.0
	30%					157.5	67.5
	40%					135.0	90.0

**Table 8:** Mix proportion of Concrete.

Test ID	W/B (%)	Unit Weight (kg/m^3^)						
		OPC	GGBS	Fine Aggregate	Coarse Aggregate	Water	Spent CO_2_ Absorbent (aq.)	Ad.
Plain	48.6	174	174	727	1015	174	-	0.8
CO_2_-10%						156.6	17.4	0.92
CO_2_-20%						139	34.8	0.98

## Data Availability

The original contributions presented in this study are included in the article. Further inquiries can be directed to the corresponding author.
